# Modelling ocean acidification effects with life stage-specific responses alters spatiotemporal patterns of catch and revenues of American lobster, *Homarus americanus*

**DOI:** 10.1038/s41598-021-02253-8

**Published:** 2021-12-02

**Authors:** Travis C. Tai, Piero Calosi, Helen J. Gurney-Smith, William W. L. Cheung

**Affiliations:** 1 Changing Ocean Research Unit, Institute for the Oceans and Fisheries, The University of British Columbia, 2202 Main Mall, Vancouver, BC V6T 1Z4 Canada; 2 Départment de Biologie, Chimie et Géographie, Université du Québec à Rimouski, 300 Allée des Ursulines, Rimouski, QC G5L 3A1 Canada; 3 Fisheries and Oceans Canada, St. Andrews Biological Station, 125 Marine Science Drive, St. Andrews, NB E5B 0E4 Canada

**Keywords:** Ecology, Zoology, Ecology, Environmental sciences, Environmental social sciences

## Abstract

Ocean acidification (OA) affects marine organisms through various physiological and biological processes, yet our understanding of how these translate to large-scale population effects remains limited. Here, we integrated laboratory-based experimental results on the life history and physiological responses to OA of the American lobster, *Homarus americanus*, into a dynamic bioclimatic envelope model to project future climate change effects on species distribution, abundance, and fisheries catch potential. Ocean acidification effects on juvenile stages had the largest stage-specific impacts on the population, while cumulative effects across life stages significantly exerted the greatest impacts, albeit quite minimal. Reducing fishing pressure leads to overall increases in population abundance while setting minimum size limits also results in more higher-priced market-sized lobsters (> 1 lb), and could help mitigate the negative impacts of OA and concurrent stressors (warming, deoxygenation). However, the magnitude of increased effects of climate change overweighs any moderate population gains made by changes in fishing pressure and size limits, reinforcing that reducing greenhouse gas emissions is most pressing and that climate-adaptive fisheries management is necessary as a secondary role to ensure population resiliency. We suggest possible strategies to mitigate impacts by preserving important population demographics.

## Introduction

Ocean acidification (OA) and climate change drivers are affecting marine environments, altering species’ performances, distribution and abundance^1^, and in turn the supply and access to marine resources that we depend on for food, livelihoods, and economic development^2–4^. While these global changes pose risks to society’s dependence on marine fisheries resources, management strategies to reduce pressures on fisheries stocks could mitigate negative effects of global ocean changes^5–8^. Understanding how marine organisms respond to multiple stressors, shift their geographic distribution and change their local abundance is essential to manage added pressures of fishing.

Research on OA effects has significantly increased in the past two decades, fundamentally improving our understanding of its direct and indirect impacts on marine life^9–11^. Despite the diversity of responses to OA, we have identified organisms that are likely to be most sensitive to changes in ocean pH. Shell forming organisms—such as corals, mollusks, and crustaceans—appear particularly sensitive to changes in ocean chemistry linked to OA, as they can be severely impacted by high dissolution rates and increased energy costs linked to the need to increase the effort for homeostasis and mineralisation to form and maintain calcium carbonate shell components^12,13^. Organisms at early stages in their life cycle (i.e. larval and juvenile) appear to be more sensitive to OA^14–20^. In species with complex life history traits, such as marine invertebrates, different life stages represent a sequence of biotic filters^21^ that are expected to have life-stage specific sensitivity to OA. These biotic filters cumulatively affect species’ recruitment and survival.

Implications of OA and climate change present new and complex challenges for fisheries. These phenomena together increase the uncertainty for fisheries as rapidly changing environmental conditions drastically affect seasonal recruitment of many stocks^22^. This in turn increases the risks to the livelihoods of > 260 million people that depend on marine capture fisheries as a source of employment^23,24^. Ocean acidification is expected to have the greatest effects on invertebrates^12,25^, which will affect global shellfish fisheries, some of the most valuable fisheries in the world valued at over 50 billion USD and one third of the value of internationally traded seafood^26,27^. Furthermore, many developing nations rely on invertebrates as an important ‘backup’ source of nutrition, and account for up to 50% of animal protein intake in some countries (e.g. Fiji)^28,29^.

In this study, we use a multi-disciplinary approach to link biological responses to global change drivers to downstream effects on fisheries using the American lobster, *Homarus americanus* (H. Milne-Edwards), as our model organism, within the context of the Canadian fisheries. Canada’s lobster fishery is one of the most economically lucrative fisheries, valued at over $1.6 billion CAD annually and further contributes over $2 billion CAD to the Canadian economy as exports^30^. First, we integrated experimental evidence of biological responses to OA amongst other stressors (i.e. ocean warming, de-oxygenation) to model future global change effects on various life stages of *H. americanus*. We explored the added effects of OA on mortality of larval, juvenile and adult stages. Next, we model these changes in a dynamic bioclimatic envelope model (DBEM) to observe changes in distribution, abundance, and fisheries catch. Lastly, we explored various broad fisheries management measures—specifically fishing pressure and size limits—that affect the dynamics of populations and the sustainability of fisheries. We tested the sensitivity of our model to uncertainties surrounding the structure and parameter selection. This is a continuation of the development of integrating OA effects into a dynamic bioclimatic envelope model^31,32^ to improve our understanding of OA effects on population dynamics and fisheries in a multi-stressor framework, and advance the general application of these models to broad scale fisheries analyses.

## Results

**Figure 1:**
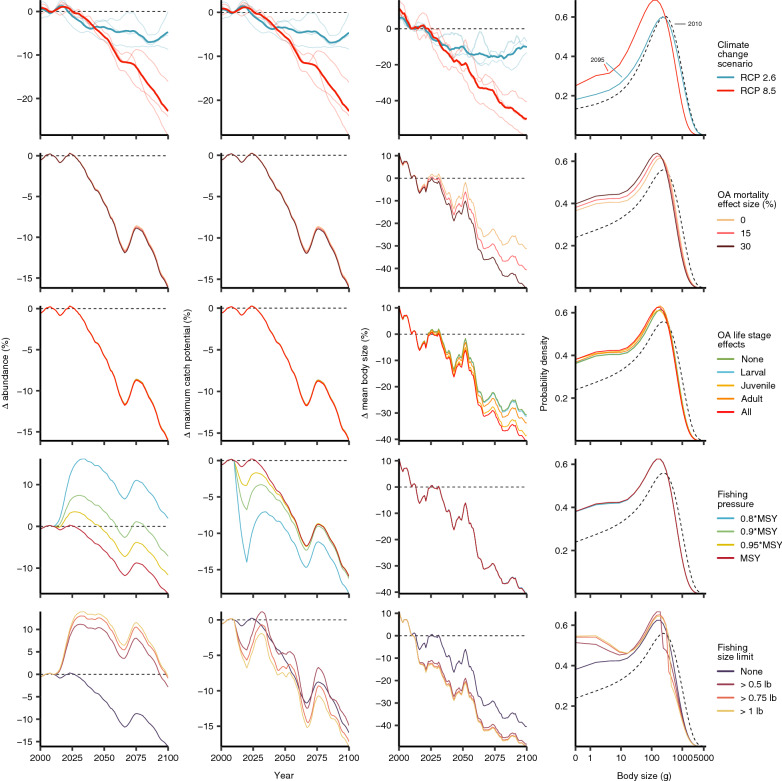
Alternative model parameter settings and their effects on abundance, maximum catch potential, and body size for the American lobster, *Homarus americanus*. While exploring change for each parameter, other parameters were held constant, such that: earth system model = GFDL; climate change scenario = RCP 8.5; OA effect size = 15%; OA effects on life stage = all; fishing pressure = MSY; fishing size limit = none. Dashed lines are the relative value in 2010, while coloured lines are temporal trends in the first three columns, and a snapshot of the population’s body size distribution in year 2095 in the far-right column. Values are relative to 2010 and are smoothed with 10-year running means.

Global ocean changes (including ocean warming and ocean acidification) are projected to have negative effects on the abundance and maximum catch potential (MCP) of the American lobster, with elevated impacts (more than 20% decrease in abundance and MCP) when climate change is accelerated (RCP 8.5; Fig. 1). Even with strong mitigation of carbon emissions (RCP 2.6), abundance and maximum catch potential are projected to decrease by up to 10% by 2100 relative to 2010. Mean body size of lobsters is also projected to decrease by 10 and 45% by the end of the twenty-first century for RCP 2.6 and RCP 8.5, respectively.

Changes in OA effects on mortality had very small effects overall on abundance and catch potential. However, there was a clear negative relationship with OA and mean body size of the population—mean body size decreased by an additional 10% by the end of the century under a high climate change scenario when OA effect size (see methods) was 15%, and another 10% decrease when the effect size was doubled (Fig. 1). When considering the effects of OA on mortality for various life stages, the effects on the juvenile stages had the greatest impacts on mean body size at the population level, suggesting that population demographics are most sensitive to impacts on juveniles.

When fishing rate is reduced, there were initial increases in abundance and catch potential as expected. At the lowest fishing level explored (i.e. assuming fishing at 0.8 times the maximum sustainable yield or MSY), abundance initially increased by 15% (Fig. 1), but then decreased by 20% to a change of − 5% by the end of the twenty-first century due to environmental stressors, relative to 2010 under RCP 8.5. Alternatively, fishing at the long-term sustainable level (i.e., assuming fishing at MSY) under RCP 8.5 resulted in an overall decrease of 20% in abundance by 2100. Furthermore, scaling back fishing at 0.8 times the MSY resulted in an initial decrease in catch potential, but was only 2% lower than fishing at MSY by the year 2100 under RCP 8.5.

Limiting the minimum size that the fisheries could target had a greater effect on abundance, but much smaller effect on catch potential (Fig. 1). While, implementing body size limits considerably increased overall abundance and reduced climate effects on abundance, over time there was no projected benefits to overall catch potential compared to the no fishing size limit scenario. Implementing fishing size limits had a large effect on the mean body size compared to no size limit, but minimal effects at different levels of size limits. Smaller mean body sizes with the implementation of size limits resulted from a relative increase in smaller individuals as larger individuals were removed from the population. Current fishing size limits vary based on region—more northern regions (e.g. Northumberland Strait) have smaller size limits—and are based on length, resulting in canner-sized lobsters (0.5–1 lb; market-sized lobsters are greater than 1 lb). We used weight for size limits to streamline our analyses for the two lobster market size classes.

**Figure 2:**
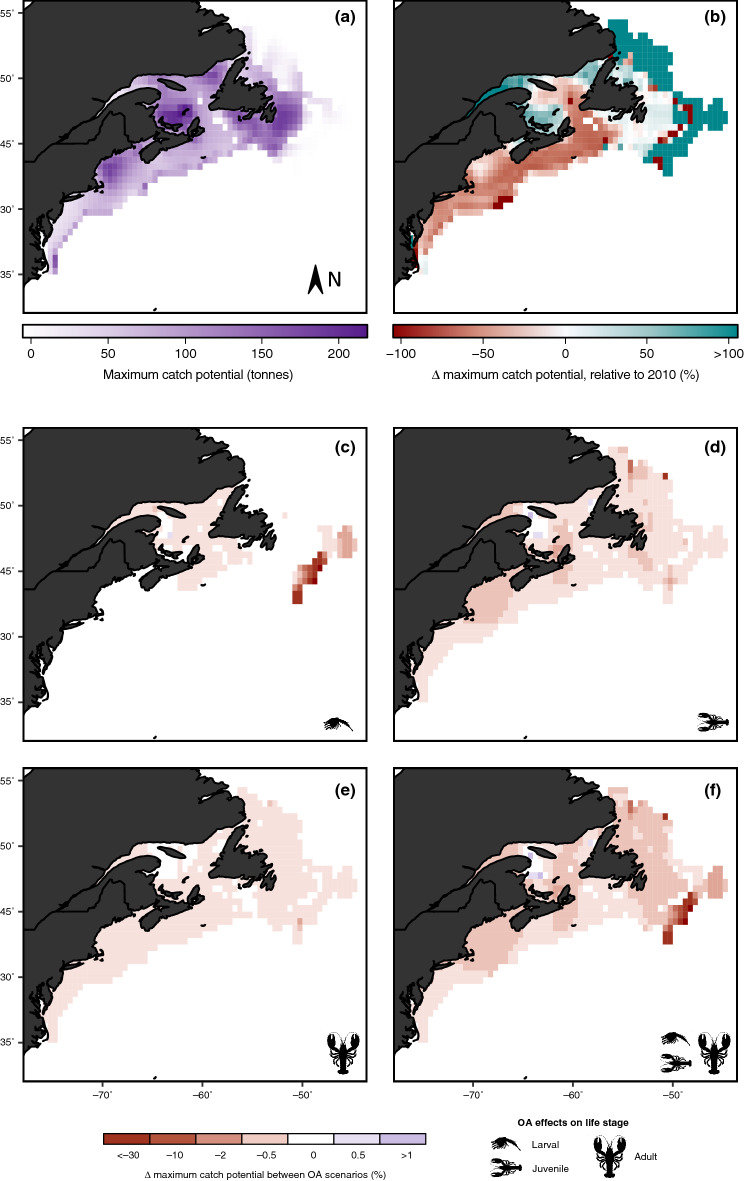
Effects of environmental change on the distribution of maximum catch potential for *H. americanus* at the end of the twenty-first century (2091–2100) in a high climate change scenario (RCP 8.5). The top two panels show the null model effects of environmental change without any OA effects (i.e. changes due to temperature, oxygen content, primary production): (**a**) estimated maximum catch potential in year 2100, and (**b**) the change in maximum catch potential relative to 2010. Panels (**c**)–(**f**) represent the change in catch potential due to the added effects of OA, relative to the null model (panel **a**), on the different life stages (larval, juvenile, adult). Results are from simulations using the GFDL Earth system model and are the annual average across 10 years. Other parameter values (Table 1): OA mortality effect size = 15%; fishing pressure = MSY; fishing size limit = none.

Projections of high climate change (including all global change drivers) on MCP show that future catch is expected to remain on continental shelf areas, but with much less biomass at lower latitudes (Fig. 2a). Changes in MCP show a shift in distribution poleward (north), and losses at lower latitudes (south) under a high climate change scenario (Fig. 2b).

The effects of OA on MCP show geographical differences based on which life history stage we model OA effects on. Ocean acidification effects on early life stages (larvae and juvenile) affect the ability for the population to shift their distribution to new, more favourable habitats (Fig. 2c,d), while OA effects on adult size classes show more homogenous effects across the entire range (Fig. 2e). Early life stages are known to be more sensitive to environmental stressors^18–20^, and our results show that OA can have major implications on range shifts. Specifically, cumulative OA effects on all life stages of lobster may restrict migration to suitable habitat as well as reduce abundance across the entire distribution (Fig. 2f).

**Figure 3:**
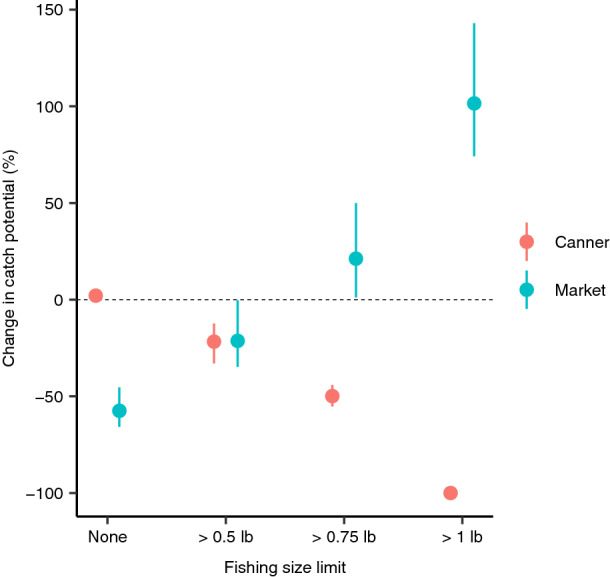
The effect of implementing different fishing size limit on change in maximum catch potential for the two market size categories of *H. americanus* by end of twenty-first century (2091–2100, relative to 2010) under a high climate change scenario (RCP 8.5), and the effects of implementing fishing size limits. Points represent multi-model means from simulations from three Earth system models and error bars represent the minimum and maximum of the three outputs. Null parameter values were used (Table 2): climate change scenario = RCP 8.5; OA mortality effect size = 15%; OA effects on life stage = all; fishing pressure = MSY.

Interestingly there are considerable differences in the effects of fishing body size limits for the two lobster market size classes. While canner-sized lobsters see no change in catch potential with no fishing size limit under a high climate change scenario by year 2100, catch potential for market-sized lobsters decreased by almost 60% (Fig. 3), averaging out to the overall decrease in catch potential of ~ 15% (Fig. 1). Implementing and increasing size limits results in decreased catch potential for canner-sized lobsters, while market-sized lobsters showed increases in catch potential (Fig. 3)—an increase of > 100% in catch potential of market-sized lobsters (relative to current catch of market-sized lobsters) with the largest fishing size limit of 1 lb. However, the combined overall (by biomass) change in catch potential was negligible with implementation of increasing size limits (Fig. 1).

**Figure 4:**
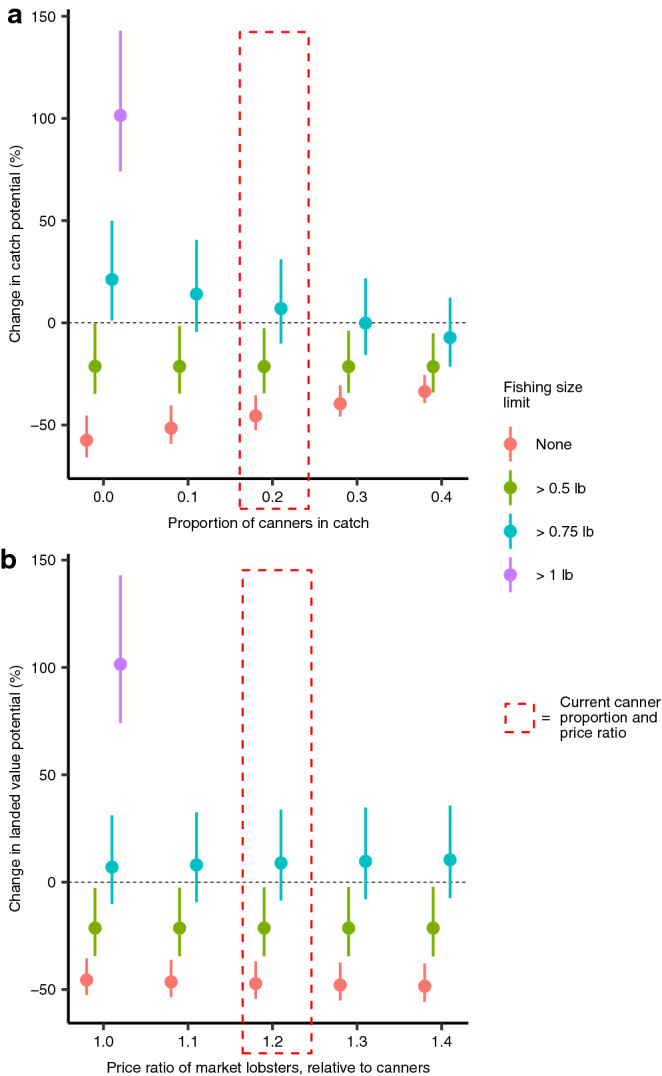
The effect of implementing different scenarios of fishing size limits on: (**a**) maximum catch potential based on different catch proportions of canner lobsters, and (**b**) landed value potential with different price ratios of market to canner lobsters. Values are the change weighted by tonnage of the different priced lobster sizes by end of twenty-first century (2091–2100) under a high climate change scenario (RCP 8.5), relative to 2010 for American lobster biomass. Proportion of canners in catch were kept constant at 0.2 (the current value) for the analysis in panel b, except for when size limits were > 1 lb (thus no canners in catch). Points represent multi-model means from simulations from three earth system models and error bars represent the minimum and maximum of the three outputs. Parameter values were used from our null model (Table 2): climate change scenario = RCP 8.5; OA mortality effect size = 15%; OA effects on life stage = all; fishing pressure = MSY.

Continuing with a high climate change scenario, there is a positive relationship with an increase in fishing size limits on catch potential by the end of the twenty-first century, regardless of the proportion of canner-sized lobsters in catch (Fig. 4a). However, the margin of increase in catch potential due to fishing size limits lessens if the proportion of canners in fishing catch increases (Fig. 4a). The large increase in catch potential for fishing size limits set at > 1 lb is exclusively due to an increase in market-sized lobsters (Fig. 3). Price differences between canner- and market-sized lobsters have a very minimal effect on the landed value (Fig. 4b). Nonetheless, restricting fishing limits to > 1 lb increases the abundance of market-sized lobsters (Fig. 3), increases their catch potential (Fig. 4a), and increases the landed value (Fig. 4b). Our results here show that fishing size limits can have considerable benefits for mitigating OA and climate change impacts.

## Discussion

The American lobster will likely be affected by projected climate change scenarios, and while OA shows little impacts to overall abundance, it may have major implications for population demographics, as already observed in systems that are naturally characterised by high CO_2_ levels^33,34^. Previous research on crustaceans, including the American lobster, has shown that individuals in the juvenile stage may be most sensitive to OA^18–20,35–37^, and our results further support this notion, suggesting that lobster populations are most sensitive to OA during juvenile stages with resulting impacts on mean body size in the population (Fig. 1). Geographical responses of the population are most sensitive when OA affects larval and juvenile stages (Fig. 2c,d). Furthermore, our findings of poleward shifts in distribution due to ocean changes are consistent with changes observed (e.g. lobster nurseries off the East coast of the US^38^)^39–41^. Recent stock assessments show that lobster abundance and landings from the Gulf of Maine/Georgia Bank area has stabilized in recent years while those from southern New England has been decreasing in recent decades (ASMFC 2020). However, other evidence points to the ongoing and future (probably irreversible) decline of the lobster population in the Gulf of Maine^42^. Our model projects that these trends may continue with declines after recent periods of more stable abundance and landings in the Gulf of Maine/Georgia Bank area, while those in the southern New England stock may continue to decline. In the Maritime estuary of the St. Lawrence River, the American lobster has made its appearance in recent years and has increased considerably its abundance in the last decade along the North coast of the Gulf of the St. Lawrence (Bernard Saint-Marie, *pers. comm*.^†^). However, despite the projected increase in lobster abundance in portions of the Maritime Estuary and Gulf of St. Lawrence (Fig. 2b), the continuation of such increasing trends in the future is more uncertain as our results show that ocean conditions could change beyond the levels that are physiologically optimal for the growth and survival of lobster in certain areas of the Gulf. There are also model limitations due to local factors that may not accurately estimate projected habitat suitability and abundances; e.g. projected changes in lobster catch potential in specific regions of the St. Lawrence system may not occur as the consequence of an intermediate cold layer in the water column which prevents larval settlement, recruitment and ultimately establishment of viable lobster populations. It is important to note that lobster populations are currently absent in these areas. Therefore, our model appears to overestimate present and future habitat suitability—and catch potential in some regions—and future models may need to downscale to fisheries management operational scales. Continuous monitoring of the biogeography of the lobster, combined with physiological and modelling experiments could further elucidate the interactions between changing ocean conditions on the future trajectory of lobster in different parts of their range. Importantly, our results underscore the importance of considering the variation in sensitivity to global ocean change across multiple life stages^43^. While our modelling of life stages may be simplistic due to the limited knowledge available from current empirical studies, it nonetheless offers valuable insights on how life-stage dependent sensitivities to changes in ocean conditions will affect the impacts of global change on exploited populations and dependent fisheries, especially for species—such as lobsters—with complex life-history strategies.

The impacts of climate change and OA on lobster populations from our models underscore the vulnerabilities of valuable commercial species and its fisheries to global environmental changes^5^. Our results show that implementing even simple changes to fisheries management and regulation can mitigate OA and climate change effects at the population level. Changes to fishing mortality rates could mitigate the effects of climate change by increasing the overall population biomass. However, catch potential is reduced with a reduced harvest rate, even when abundance increases. Therefore, a dynamic harvest strategy would be needed to offset the initial reductions in catch potential from a lower harvest rate once overall abundance shows improvement (i.e. population growth). Harvest-control conservation efforts have shown to sustain populations and boost fisheries production in the eastern US and could act as an effective mitigation strategy against climate change impacts^44^. Our results further corroborate this idea, and show that initial reductions in catch can boost population abundance whilst not forgoing much change in long-term catch potential. While we did not test implementing maximum fishing size limits; returning larger and more fecund individuals could further mitigate climate change impacts on the populations^44^.

There are considerable uncertainties associated with the quantitative projection for the key model indicators presented here. As highlighted in Cheung et al. (2016), such uncertainties are caused by the coarse resolution of the ocean conditions projected by the Earth system model used in this study, particularly for species that occur mostly along shelf seas. Also, the model assumes that the initial species distribution is in equilibrium with historical environmental conditions, which might not be the case. Next, this study did not explicitly account for the effects of trophic interactions^21^ and the potential for the species to adapt through evolutionary responses^43^. While our modelling of OA and climate-related impacts on lobsters may have been simplistic, our study provides a foundation for future studies to build on, for example, by using outputs from high resolution regional oceanographic models, more explicitly accounting for trophic interactions, incorporating adaptive potential through microevolutionary responses, integrating interactive effects of multiple stressors and filling data gaps on cumulative (including disease and pathogens) and carry over impacts (across life stages and successive generations) on population mortality and reproductive output. These could be done by either making DBEM more complex by adding these biological components into the model as data becomes available, or using alternative modelling approaches. The latter has the added benefits of providing additional sets of model outputs to explore more structural uncertainties^45,46^. However, even with simplistic assumptions of life history stages, our model showcases the importance to consider the variety of responses that a species can display across its developmental trajectory, the various responses of populations to climate and anthropogenic courses of actions, and a model representation for a general approach to fisheries sustainability and climate change mitigation.

This modelling exercise projected scenarios of changes in the sustainability of American lobster fisheries under climate-related impacts (e.g. OA, ocean warming) and fisheries management options. Implementation of fishing size limits can also help population abundance against climate change and OA without large changes to catch potential. Where fishing size limits may have the greatest effect is on the proportion of canner and market sized lobsters and the weighted landed values (Figs. 3 and 4). Growth overfishing—when individuals are harvested at sizes below that would produce MSY *per* recruit—has occurred in almost all American lobster fisheries^47^, and support our results that suggest that changes in size limits can benefit the population and fisheries catch. Particularly, the comparisons of the projected outputs across scenarios offer useful insights into the potential sensitivities of the American lobster fisheries to fishing and CO_2_-related hazards. While these fishing management scenarios in our model are broad, they provide potential strategies for climate change mitigation. Extreme shifts in geographic distribution and the available suitable lobster habitat can also limit the capacity for compensation for any changes in fisheries management to mitigate climate impacts on localized populations. Therefore, more localized and dynamic (spatially and temporally) management strategies could be tested in future studies, especially to better align with current management regimes across the region. However, adaptation strategies to offset climate-related impacts through fisheries management are limited^6,8^, especially under the high climate change scenario, where increased effects of climate change later in the twenty-first century outweigh the reductions in fishing mortality; this strongly suggests that greenhouse gas emissions reductions are the key factor for reducing impacts and increasing the mitigative potential of changes in fisheries management. Therefore, the greatest efforts to reduce impacts on lobster populations, its fisheries associated livelihoods and economies will be through climate change mitigation and keeping global warming below + 1.5 °C relative to pre-industrial level —as close to the optimistic RCP 2.6 scenario as possible. Governments need to commit to setting the course to meet these targets in the very near term. Nonetheless, there are opportunities for climate adaptation through fisheries management measures.

## Methods

### Model species

The American lobster *Homarus americanus*, is a crustacean found in temperate regions across the Northwest Atlantic Ocean. It is a highly valuable fishery species, caught off the coast of Eastern Canada and Northeast USA. For the past decade, they have been the most valuable single-species fishery in all of Canada and the US^30,48^. In Canada, catch was estimated at 104,000 tonnes and 14% of all of Canada’s total marine fisheries catch in 2019. However, their landed value was almost $1.6 billion and 44% of the Canada’s total marine fisheries landed value, and over 49% of the landed value from Canada’s Atlantic coast fisheries^30,49^. Canada’s Atlantic marine fisheries provide employment to over 40,000 people in primary harvesting and processing sectors, and supports many rural populations^30^.

### Dynamic bioclimate envelope model

We used a dynamic bioclimate envelope model (DBEM)^31,50^ to assess spatial and temporal changes in the abundance and fisheries catch under different scenarios of climate change and fishing pressure. The model infers the environmental preference of the modelled species^51^, and simulates future changes in biomass and maximum catch potential. Notably, the DBEM integrates growth models^52^, ecophysiological models^53^, advection–diffusion models^54^, and surplus production population dynamics models^55^ to determine ocean change effects on species distribution, abundance, and catch. Since *H. americanus* is primarily a benthic invertebrate, we used values at sea floor for environmental variables in our model. However, they also have a pelagic larval phase and we used surface environmental variables to model this life stage.

#### Initial distribution and abundance

The DBEM uses an initial species distribution determined using a rule-based algorithm^56–58^. This algorithm determines species’ distribution range base on a series of geographic constraints, including latitudinal range, depth range, occurring ocean basins, and published or expert provided ‘bounding box’. It assumes that the relative abundance of the species distributes along gradients within these geographic limits with the centroid of the range having the highest relative abundance (Palomares et al. 2016). Species distributions are mapped on a global grid with a resolution of 0.5° longitude by 0.5° latitude cells with values representing relative abundance. Historical reconstructed catch data (http://www.seaaroundus.org)^59^ was used to estimate the global abundance and distributed accordingly with relative abundance values^60^. These catch data are based on various government and non-government reports, primary and grey literature, and also mapped on a 0.5° longitude by 0.5° latitude grid.

The initial species distribution is then overlaid on climatologies of historical environmental conditions (e.g. temperature, salinity, oxygen, pH) simulated from outputs of Earth system models (e.g. Bopp et al. 2013; Dunne et al*.* 2013). The DBEM assumes that species distribution is in equilibrium with the average historical environmental condition (1971–2000 average) and abundance in cells are assumed to be at carrying capacity.

#### Modelling individual growth

Growth is modelled using a derived von Bertalanffy growth model to incorporate how environmental stressors affects body size of lobsters^31,32,61^. We model the following important life history parameters as a function of relative changes in temperature, oxygen content [O_2_], and pH [H^+^]. Growth rate (*dB*/*dt*) is dependent on weight-specific anabolic and catabolic rates:

1 $$\frac{dB}{{dt}} = H_{i,t} W^{d} - k_{i,t} W$$

where *H* and *k* represent the coefficients for oxygen supply (anabolism) and oxygen demand for maintenance metabolism (catabolism), respectively, for cell *i* at time *t*. Body weight is scaled to anabolism with the exponent *d* < 1 while it is scaled linearly with catabolism. Values of *d* typically fall between 0.5 and 0.95 across invertebrate species, and we assume *d* = 0.7 for our analysis on *H*. *americanus*. Other values of *d* have been tested in previous studies; larger values resulted in much higher sensitivity to environmental stressors, while smaller values resulted in a minimal decrease in sensitivity^32,61^. We can rearrange Eq. (1) and solve for *H* when growth rate, *dB/dt* = 0 and maximum body size *W*_*∞*_ is reached, such that:

2 $$H_{i,t} = k_{i,t} W_{\infty ,t}^{(1 - d)}$$

We use parameter values from SeaLifeBase (http://www.sealifebase.ca)^62^ for maximum body length, *l*_*∞*_, and growth rate, *K*, from the von Bertalanffy growth equation, $l_{t} = l_{\infty } (1 - e^{{ - K(t - t_{0} )}} )$, where *l* is the length and *t* is the age in years (Table 1)^63^. Maximum body weight, *W*_*∞*_, is calculated using the length–weight conversion equation, $W = al^{b}$, where *a* and *b* are coefficients also taken from SeaLifeBase (Table 1). Growth rate, *K*, is related to catabolic coefficient *k*:

3 $$K = k(1 - d)$$

**Table 1:** Initial parameter values for *Homarus americanus*. *l*_*∞*_ = maximum length; *W*_*∞*_ = maximum weight; *K* = von Bertalanffy growth parameter K; *a* = length–weight relationship parameter a; *b* = length–weight relationship parameter b; *MaxD* = maximum depth; *MinD* = minimum depth; *r* = intrinsic population growth rate.

$l_{\infty ,0}$(cm)	$W_{\infty ,0}$(g)	$K_{o}$(year^−1^)	*a*	*b*	*MaxD *(*m*)	*MinD *(*m*)	*r* (year^−1^)
64	2621	0.1	0.01	3	500	1	0.2

Parameters *H* and *k* were estimated as a function of environmental stressors:

4 $$H_{i,t} = g_{i} \left[ {{\text{O}}_{2} } \right]_{i,t} {\text{e}}^{{ - j_{1} /T_{i,t} }}$$

and

5 $$k_{i,t} = h_{i} \left[ {{\text{H}}^{ + } } \right]_{i,t} {\text{e}}^{{ - j_{2} /T_{i,t} }}$$

where $e^{ - j/T}$ represents the Arrhenius equation to model the change in chemical reactions as a function of temperature *T* in degrees Kelvin. The parameters *j* are equal to *E*_*a*_/*R* where *E*_*a*_ is the activation energy and *R* is the Boltzmann constant, respectively; activation energies are estimated to be 0.388 eV and 0.689 eV, based on Cheung et al*.* (2011), resulting in a *j*_*1*_ and *j*_*2*_ of 4500 K and 8000 K, respectively. Coefficients *g*_*i*_ and *h*_*i*_ are fixed parameters throughout the simulation and estimated by rearranging Eqs. (4) and (5), and substituting Eqs. (2) and (3) for *H* and *k*:

6 $$g_{i} = \frac{{W_{\infty ,0}^{(1 - d)} k_{0} }}{{\left[ {{\text{O}}_{2} } \right]_{0,i} e^{{ - j_{1} /T_{0} }} }}$$

and

7 $$h_{i} = \frac{{K_{0} /\left( {1 - d} \right)}}{{\left[ {H^{ + } } \right]_{0,i} e^{{ - j_{2} /T_{0} }} }}$$

given initial values of maximum body size *W*_*∞,0*_ and von Bertalanffy growth parameter *K*_*0*_, and initial environmental conditions of temperature, oxygen concentration, and hydrogen concentration.

We measured impacts of changes in environmental conditions on growth by estimating new *H*_*i,t*_ and *k*_*i,t*_ coefficients using Eqs. (4) and (5). Equation (2) can be rearranged to solve for a new maximum body size *W*_*∞*_ using new values of *H*_*i,t*_ and *k*_*i,t*_, while Eq. (3) can be used to calculate a new growth parameter *K*.

To simulate change in mean body size using DBEM, we used a size transition matrix, *X*, to model the probabilities of an individual growing from one length class to other size classes in one time-step (year) and each grid cell where the American lobster was predicted to occur^50,64^:

8 $$X_{{i,t,l^{\prime},l}} = \frac{{\theta_{{y,i,t,l^{\prime},l}} }}{{\mathop \sum \nolimits_{l} \theta_{{y,i,t,l^{\prime},l}} }}$$

and

9 $$\theta_{{y,i,t,l^{^{\prime}} ,l}} = \exp \left[ { - \frac{{\left( {l - \left[ {l_{\infty ,i,t} \left( {1 - e^{{ - K_{i,t} }} } \right) + l^{\prime}e^{{ - K_{i,t} }} } \right]} \right)^{2} }}{{2\sigma^{2} }}} \right]$$

where *l* and *l*′ are the length of a particular size class and the adjacent length size classes, *l*_*∞*_ is the asymptotic length, *y* is the age of an individual, and *K* is the von Bertlanffy growth parameter. Variation in growth, σ, assumed to have a coefficient of variation of 20% and is independent of length and age^50^. Our model applies this general size transition model and makes no assumptions of growth stages specific to lobsters (e.g. moulting) or sex.

Mean body size (g), $\overline{W}$, is calculated:

10 $$\overline{W}_{i,t} = \frac{{\mathop \sum \nolimits_{y} \mathop \sum \nolimits_{l} W_{l} \cdot X_{{i,t,l^{\prime},l}} \cdot S_{i,t,y - 1,l} \cdot e^{{ - M_{i,t} }} }}{{\mathop \sum \nolimits_{y} \mathop \sum \nolimits_{l} X_{{i,t,l^{\prime},l}} \cdot S_{i,t,y - 1,l} \cdot e^{{ - M_{i,t} }} }}$$

where *S* is a relative distribution length-age frequency matrix from age class $y - 1$ at size class *l*, and initial relative distribution at age 0 (when *y* = *1*) across length classes was assumed to be $S_{i,t,0,l} = 1\;\;0\;\;0 \;\; \ldots \;\;0_{{l_{\infty ,i,t} }}$. Parameter *M* is the population natural mortality, calculated from maximum body size *W*_*∞*_, von Bertalanffy growth parameter *K*, and temperature *T*_*Celsius*_ (in degrees Celsius) using a model developed by^52^:

11 $$M_{i,t} = - 0.4851 - 0.0824\log (W_{\infty ,i,t} ) + 0.6757\log (K_{i,t} ) + 0.4687 \log (T_{Celsius,i,t} )$$

Spawning biomass is estimated using the size transition matrix, *X*, and the mean weight of each size class for size classes greater than the size at maturity, *l*_*mat*_^65^:

12 $$l_{mat,i,t} = l_{\infty ,i,t} \left( {0.714} \right)^{1/(1 - d)}$$

Length at maturity is determined for each cell based on the maximum body size *l*_*∞*_ as determined by Eq. (2) and the length–weight conversion equation.

### Modelling population biomass

Population biomass is modelled using a logistic growth model^55^, where biomass (*B*) can converted to relative abundance (*A*) using mean weight ($\overline{W}$) with the formula $A_{i,t} = B_{i,t} /\overline{W}_{i,t}$. Thus abundance in cell *i* between time *t* and *t* + *1* was estimated using:

13 $$A_{i,t + 1} = A_{i,t} + rA_{i,t} \left( {1 - \frac{{A_{i,t} }}{{C_{i,t} }}} \right) + \mathop \sum \limits_{j = 1}^{N} \left( {L_{j,i,t} + I_{j,i,t} } \right) - A_{i,t} \left( {1 - e^{{ - (F_{i,t} + \overline{M}_{i,t} )}} } \right)$$

where *r* is the intrinsic population growth rate of the species (Table 1), *C*_*i,t*_ is the carrying capacity for each cell *i*, $L_{j,i,t}$ and $I_{j,i,t}$ are the settled larvae and net migrated adults, respectively, into cell *i* from surrounding cells *j*, and *F*_*i,t*_ is the fishing mortality rate. Average mortality, $\overline{M}$, for each cell was weighted by size class specific mortality rates tested in this study. Grid cells are assumed to be at carrying capacity from the start of the simulation, and carrying capacity changes as a function of habitat suitability, *P*, and primary production, *PP*, from initial conditions (*t* = 0) to the current timestep, *t*, such that:

14 $$C_{i,t} = C_{i,0} \cdot \frac{{P_{i,t} }}{{P_{i,0} }} \cdot \frac{{PP_{i,t} }}{{PP_{i,0} }}$$

Habitat suitability is dependent on five environmental factors in combination with species specific traits, such that:

15 $$P_{i} = P\left( {T_{i} , \;TPP} \right) \cdot P\left( {Bathy_{i} , \;MinD, \;MaxD} \right) \cdot P\left( {Habitat_{i,j} , \;HAssoc} \right) \cdot P\left( {Salinity_{i} , \;SAssoc} \right) \cdot P\left( {Ice_{i} , \;IceP} \right)$$

Habitat suitability is determined by: *T* is temperature (Kelvin) and *TPP* is the species’ temperature preference profile; *Bathy* is the bathymetry and *MinD* and *MaxD* is the minimum and maximum depth of the species range; *Habitat*_*i,j*_ is the proportion of total area of a cell with a specific habitat *j* (e.g. inshore, offshore, coral, estuarine, etc.); *Salinity* is the salinity class of the cell based on Thalassic series—metahaline (> 40 ppt), mixoeuhaline (> 29 ppt), polyhaline (> 18 ppt), mesophaline (> 5 ppt), oligohaline (> 0 ppt)—and *SAssoc* is the association of the species with each salinity class; and *Ice*_*i*_ is the sea ice % area coverage in a cell and *IceP* is the ice-dependency of the species. For *H. americanus*, they are not specifically associated with any habitat and thus only restricted by depth parameters. However, they are limited to mixoeuhaline and polyhaline salinities^62^.

The *TPP* was estimated using the initial predicted relative abundance (described above) overlaid with the inputs of earth system models of initial environmental conditions. The relative weight for each temperature class *z* of the temperature preference profile was calculated as $TPP_{z} = R_{z} /\sum R_{z}$, where *R*_*z*_ is the relative abundance in each temperature class.

A fuzzy logic model was used to model the movement between neighbouring cells based on differences in habitat suitability^50^. Emigration into a cell is favoured if habitat suitability is higher than surrounding cells, and immigration out of a cell is favoured if habitat suitability is lower than surrounding cells.

We estimated larval production as 30% of spawning population biomass for each cell *i*, while larval mortality was 0.85 day^−1^ and settlement rate was 0.15 day^−1^—these values were chosen based on the sensitivity testing of these parameters^50^.

Larval dispersal is modelled using an advection–diffusion^54^ and a larval duration model based on temperature^66^, such that abundance *A*_*i,t*_ in each cell is numerically solved for using the equation:

16 $$\frac{{\partial A_{i,t} }}{\partial t} = \frac{\partial }{\partial x}\left( {D_{i,t} \frac{{\partial A_{i,t} }}{\partial x}} \right) + \frac{\partial }{\partial y}\left( {D_{i,t} \frac{{\partial A_{i,t} }}{\partial y}} \right) - \frac{\partial }{\partial y}\left( {u \cdot A_{i,t} } \right) - \frac{\partial }{\partial y}\left( {v \cdot A_{i,t} } \right) - \lambda \cdot A_{i,t}$$

while adult dispersal is similarly modelled,

17 $$\frac{{\partial A_{i,t} }}{\partial t} = \frac{\partial }{\partial x}\left( {D_{i,t} \frac{{\partial A_{i,t} }}{\partial x}} \right) + \frac{\partial }{\partial y}\left( {D_{i,t} \frac{{\partial A_{i,t} }}{\partial y}} \right)$$

Advection was modelled for larval dispersal using parameters *u* and *v* for horizontal (east–west) and vertical (north–south) directions for surface current velocity (m^2^ s^−1^), respectively, between neighbouring cells *x* and *y* in the east–west and north–south direction, respectively. Instantaneous rate of larval mortality, *M*_*L*_, and settlement, *S*_*L*_ was integrated into Eq. (16), where $\lambda = 1 - e^{{ - \left( {M_{L} + S_{L} } \right)}}$. The coefficient *D*_*i,t*_ is the diffusion parameter:

18 $$D_{i,t} = \frac{{D_{i,0} \cdot m}}{{1 + e^{{(\tau \cdot P_{i,t} \cdot \rho_{i,t} )}} }}$$

and

19 $$\rho_{i,t} = 1 - \frac{{\emptyset_{i,t} }}{{\left( {C_{i,t} /\overline{W}_{i,t} } \right)}}$$

where *D*_*i,*0_ is the initial diffusion coefficient and a function of the spatial grid size (*GR*): $D_{i,0} = \left( {1.1 \times 10^{4} } \right) \cdot GR \cdot 1.33$. Parameters *m* and $\tau$—both set at 2 in the model—determine the curvature of the functional relationship between *D*, *P*, and $\rho$^50^. Parameter $\rho_{i,t}$ represents density-dependent factors and a function of population density (number of individuals), $\emptyset_{i,t}$, carrying capacity ($C_{i,t}$), and mean body weight ($\overline{W}_{i,t}$) in each cell *i*.

### Models of ocean acidification effects

The DBEM operates to model larval dispersal using advection–diffusion models. Survival is calculated at each time step (biweekly) based on a static annual survival rate. We recently tested a simple linear relationship between survival rate and pH, represented by percent changes in the survival rate given a change in pH^32^. We used parameters derived from previous experimental studies, where they observed a ~ 15% increase in mortality in larval and juvenile stages^37^ from a doubling of hydrogen ion concentration.

We explore the OA effects by modelling variations in life stage-specific sensitivities to OA. Larvae, juveniles, and adults are modelled based on size classes, and the weight at maturity determines the size at which juveniles transition into adults^65^. Therefore, impacts of OA on survival can be modelled for various size classes. We model the effects of OA on the three major life history stages—larval, juvenile, and adult—and use a correlative approach to link changes in ocean acidity with changes in survival.

The length transition matrix (Eqs. (8) and (9)) is split up into 40 length size classes, divided evenly from 0 to $l_{\infty ,i,t}$. We assume larvae transition from the pelagic phase to the growth transition matrix, and enter as the ‘larval’ stage for only the first size class. Next, juvenile size classes comprise all size classes below the length at maturity, *l*_*mat*_, as determined in Eq. (12), and lobster in any size classes greater are considered adults. While our models do not incorporate lobster-specific life cycle traits (i.e. transitioning between larval stages then to juvenile stages), we use more general models that can be broadly applied to many species.

#### Modelling effects on survival

OA effects can be modelled as relative changes in survival rate for all life stages in Table 2. In other words, percent changes in acidity (*i.e.* hydrogen ion concentration) from baseline initial conditions results in a percent change in baseline survival rate. We use a model structure similar to that of previous work we have done^32^:

20 $$Surv_{t} = Surv_{init} *\left[ {1 + \left( {p*\left( {\frac{{\left[ {H^{ + } } \right]_{t} }}{{\left[ {H^{ + } } \right]_{init} }} - 1} \right)^{w} } \right)} \right]$$

**Table 2:** Scenario settings explored with model projections.

Parameters	Values/settings				
Climate change scenario	RCP 2.6	**RCP 8.5**			
OA mortality effect size	0%	**15%**	30%		
OA effects on life stage	None	Larval	Juvenile	Adult	**All**
Fishing pressure	0.8*MSY	0.9*MSY	0.95*MSY	**MSY**	
Fishing size limit	> 0.50 lb	> 0.75 lb	> 1.00 lb	**None**	

*All combinations of parameter values were explored. Bolded cells are the values that are kept constant when exploring each parameter—the ‘null’ model.

*Surv* is the survival rate per year and used here as an example but can be applied to other life histories affected by OA (e.g. growth, reproduction). Survival rate in year *t* is derived from the initial (*init*) survival rate and the relative change in [H^+^] between year *t* and initial [H^+^] conditions. Note that in our previous model, *p* represents the point value of the percent change effect size with a doubling of [H^+^]. This model utilizes single point effect size estimates that have no underlying assumed relationship between acidity and survival. In our model, we used an exponent value, *w*, equal to 1, which assumes a linear relationship^32^.

### Fishing pressure

Fishing mortality was assumed to be at maximum sustainable yield (MSY), which is the theoretical maximum biomass that can be sustainably removed from the population indefinitely. MSY is calculated using a Gordon Schaefer population growth model^67^:

$$MSY = \frac{{B_{\infty } \cdot r}}{4}$$

where *B*_*∞*_ is the population carrying capacity and *r* is the intrinsic population growth rate. We use this measure of MSY as a proxy for the maximum catch potential (MCP) into the future, thus we assume that fisheries management are optimized and operate at MSY.

Furthermore, the fishing mortality rate, *F*_*i,t*_—i.e. the annual proportion at which biomass is taken from the current population biomass—in each cell *i* at time *t* at MSY can be calculated as:

$$F_{MSY,i,t} = \frac{r}{2}$$

The fishing mortality rate was adjusted to explore scenarios of reduced fishing pressure and interactions with climate change and OA on the population dynamics of lobster. Any reductions in fishing pressure began in 2010 to represent how changes in fishing implemented now could change the state of lobster populations with the added stressors of climate change.

### Fishing size limits

Fishing size limits were set to represent management scenarios and to observe their effects on lobster populations and size distribution of the population. We set four scenarios of minimum body size restrictions of lobster catch: no limit, canner small (> 0.5 lb, 220 g), canner large (> 0.75 lb, 320), and market (> 1 lb, 430 g). Canner lobsters are smaller lobsters that are often sold at a cheaper price. They range from 0.5 to 1 lb, and are largely caught in Northumberland Strait where size limits are currently set lower due to warmer waters and smaller size at maturity^44^. For these scenarios of fishing size limits, we continue to assume catch is at maximum sustainable yield. Therefore, the same catch biomass (calculated using fishing mortality rate, F) will be the same for the various size limit restrictions, and more biomass will be taken from upper size classes where size limits are implemented. The no size limit results in fishing mortality to all size classes (including undersized lobsters).

### Climate change scenarios

We use outputs from three Earth system models for projections of various future climate change outcomes: NOAA’s Geophysical Fluid Dynamics Laboratory (GFDL-ESM), Institute Pierre Simon Laplace Climate Modelling Centre (IPSL-ESM), and Max Planck Institute for Meteorology (MPI-ESM)^68^. These models are included in the Coupled Model Intercomparisons Projection Phase 5 (CMIP5). We used two Representative Concentration Pathways (RCPs)^69^ which are greenhouse gas (GHG) concentration trajectories derived to reflect possible combinations of various socioeconomic assumptions, RCP 2.6 and RCP 8.5. The number associated with RCPs represent the radiative forcing values by the year 2100 based on greenhouse gas concentrations. RCP 2.6 corresponds with a low climate change scenario and assumes immediate mitigation of GHG emissions where annual emissions peak by mid-decade (year 2025) but is reduced considerably. This scenario is more in line with the 2015 Paris Agreement to limit warming to + 1.5 °C relative to other emissions scenarios applied in most CMIP5 Earth system models. Conversely, RCP 8.5 corresponds to a high climate change scenario and our current trajectory where we continue to use fossil fuels, have little to no change to switch to renewable energy sources, and GHG emissions continue to increase with no implementation of any mitigation action. We chose these three Earth system models as they provide sea surface and bottom layers and the full range of environmental variables required by the DBEM (i.e. sea temperature, dissolved oxygen, primary production, pH, current advection, salinity, sea ice extent) for both RCP scenarios^2^.

All statistical analyses and figures were generated using the programming software R v4.0.3^70^.
